# Dietary Interventions and Supplements for Managing Attention-Deficit/Hyperactivity Disorder (ADHD): A Systematic Review of Efficacy and Recommendations

**DOI:** 10.7759/cureus.69804

**Published:** 2024-09-20

**Authors:** FNU Abhishek, Jaikirat Singh Gugnani, Harkamalpreet Kaur, Abhiram Rao Damera, Rohan Mane, Arushi Sekhri, Gurpreet Singh, Gurnoor Kaur

**Affiliations:** 1 Medicine, Government Medical College, Amritsar, Amritsar, IND; 2 Nephrology, Government Medical College, Amritsar, Amritsar, IND; 3 Internal Medicine, Mediciti Institute of Medical Sciences, Hyderabad, IND; 4 Neurological Surgery, University of Nis, Nis, SRB; 5 Internal Medicine, Government Medical College, Amritsar, Amritsar, IND

**Keywords:** artificial food colour, attention deficit hyperactivity disorder (adhd), diet therapy, feingold diet, vitamin-d

## Abstract

Attention-deficit/hyperactivity disorder (ADHD) significantly impacts children's neurological development, behaviour, and overall well-being, affecting academic performance and social interactions. This systematic review investigates the effectiveness of dietary interventions (DASH (Dietary Approaches to Stop Hypertension) diet, Vitamin D3 supplementation, magnesium, and impact of artificial food colors and marine oils) and supplements alongside standard pharmacotherapy in managing ADHD symptoms. Adhering to Preferred Reporting Items for Systematic Reviews and Meta-Analyses (PRISMA) 2020 guidelines, we extensively searched various databases for studies published from 2016 to 2022. Out of an initial pool of 7873 records, 14 randomized controlled trials met our inclusion criteria following rigorous screening.

Our findings suggest that gluten-free diets may alleviate ADHD symptoms despite negative celiac serology, highlighting the role of non-celiac gluten sensitivity. Moreover, vitamin and mineral supplements like vitamin D and magnesium showed varying degrees of effectiveness in improving behavioural and emotional symptoms. Innovative treatments, such as combining saffron with methylphenidate and using marine oil extracts, also demonstrated potential in enhancing behaviours associated with ADHD.

The review underscores the importance of dietary approaches, such as the DASH diet and specific exclusions (e.g., a few foods diet and an oligoantigenic diet), in managing symptoms by addressing nutritional factors. Studies on probiotics and interventions targeting gut microbiota composition yielded mixed results, necessitating further exploration. Limitations include study diversity and short-term outcome assessments, cautioning against broad generalizations. Integrating personalized dietary assessments and interventions into ADHD treatment strategies could optimize therapeutic outcomes and potentially reduce reliance on pharmacotherapy alone.

In summary, this systematic review highlights the potential benefits of dietary modifications and supplements in managing ADHD symptoms. Future research should prioritize long-term efficacy, interactions with conventional medications, and personalized dietary approaches to refine ADHD treatment strategies.

## Introduction and background

Attention-deficit/hyperactivity disorder (ADHD) profoundly affects children's neurological development and behavior, significantly impacting their well-being, academic performance, and social interactions [1,2]. According to the Diagnostic and Statistical Manual of Mental Disorders, Fifth Edition (DSM-5) criteria, ADHD manifests in predominantly inattentive, hyperactive-impulsive, or combined presentations [2]. Children with ADHD often exhibit disruptive behavior, low self-esteem, and challenges in forming interpersonal relationships, which can persist into adulthood and impair daily functioning [3].

ADHD is prevalent, affecting about 5% of children and adolescents and 3% of adults [4]. Genetic factors play a substantial role, with studies indicating a significant heritable component [5,6]. Prenatal influences, such as maternal smoking, alcohol consumption, and inadequate nutrition, also contribute to the disorder's prevalence, highlighting its complex etiology [5].

Standard treatments for ADHD primarily involve pharmacotherapy with stimulant medications such as methylphenidate and amphetamines, widely recognized for their effectiveness in managing symptoms [7]. However, these medications may induce side effects, such as reduced appetite and insomnia, prompting the exploration of alternative therapies, including non-stimulant medications and behavioral parent training (BPT) [8,9].

Recent research has also explored dietary interventions as potential adjuncts to traditional treatments. Studies suggest that supplements like micronutrients, omega-3 polyunsaturated fatty acids (PUFAs), and dietary adjustments, such as eliminating artificial additives and increasing vitamin D and magnesium intake, may positively influence ADHD symptoms [10-12]. Natural compounds like Ginkgo biloba extract and saffron have also shown promising neuroprotective and therapeutic effects [13].

This paper aims to systematically review and compare the effectiveness of pharmacological treatments versus dietary interventions in managing ADHD symptoms. It aims to provide insights into dietary therapies' potential benefits and limitations as complementary or alternative approaches in ADHD management.

## Review

Materials and methods

This systematic review followed Preferred Reporting Items for Systematic Reviews and Meta-Analyses (PRISMA) 2020 guidelines in conducting and reporting its findings. We developed a precise search strategy using Medical Subject Headings (MeSH) criteria. We comprehensively analyzed ADHD and dietary therapy across multiple databases, including PubMed, Web of Science, PubMed Central (PMC), and Google Scholar, using appropriate Boolean operators (AND, OR, NOT). We selected relevant articles focusing on childhood attention deficit disorder through a structured PubMed search using the MeSH strategy, as detailed in Table 1.

**Table 1:** PubMed search results obtained using the MeSH strategy

^MeSH strategy used^	^Results obtained^
"Attention Deficit Disorder with Hyperactivity/diet therapy"[Majr] OR "Attention Deficit Disorder with Hyperactivity/drug therapy"[Majr]	^5913^

Other search engines used are listed in Table 2.

**Table 2:** Data extraction using Web of Science, Google Scholar, and PMC

^Search engine^	^Search query^	^Results obtained^
Web of Science	ALL=(ADHD AND diet therapy) OR ALL=(ADHD AND drug therapy) OR ALL=(Role of Diet therapy in ADHD)	896
Google Scholar	adhd diet	55
PMC	(("ADHD"[MeSH Terms] OR ("attention"[All Fields] AND "deficit"[All Fields] AND "disorder"[All Fields] AND "hyperactivity"[All Fields]) OR "ADHD"[All Fields] OR "adhd"[All Fields]) AND ("diet therapy"[Subheading] OR ("diet"[All Fields] AND "therapy"[All Fields]) OR "diet therapy"[All Fields] OR "diet therapy"[MeSH Terms])) AND (Randomised[All Fields] AND ("prevention and control"[Subheading] OR ("prevention"[All Fields] AND "control"[All Fields]) OR "prevention and control"[All Fields] OR "control"[All Fields] OR "control groups"[MeSH Terms] OR ("control"[All Fields] AND "groups"[All Fields]) OR "control groups"[All Fields]) AND ("clinical trials as topic"[MeSH Terms] OR ("clinical"[All Fields] AND "trials"[All Fields] AND "topic"[All Fields]) OR "clinical trials as topic"[All Fields] OR "trial"[All Fields]))	1009

The authors established inclusion criteria and selected articles based on several factors. These criteria focused on studies involving children aged 6-16 years with ADHD who received dietary interventions, publications published in English, and other specified conditions. These conditions included papers with full-text availability, studies conducting in vivo investigations, publications released within the past five years (2016-2022), and articles addressing ADHD treatment with dietary interventions. This systematic review includes randomized controlled trials (RCTs). Excluded from the analysis were articles published in languages other than English, those unrelated to the research topic, studies lacking full-text availability, and patients treated solely with other medical or psychological interventions. The extracted papers underwent rigorous quality assessment using appropriate evaluation tools for each study type. Articles with a minimum non-bias percentage of 50% were considered for inclusion after the assessment. The RCTs that met the criteria and the results of the critical appraisal using the Cochrane risk of bias tool for RCTs are given in Table 3.

**Table 3:** The results of critical appraisal using the Cochrane risk of bias tool for RCTs RCT: randomized controlled trial

^Study^	^Non-bias percentage^
Stevens AJ et al. [10]	^66.66%^
Hemamy M et al. [12]	^83.33%^
Rucklidge JJ et al. [14]	^83.33%^
Samadi M et al. [15]	^83.33%^
Honarvar NM et al. [16]	^83.33%^
Canal LA et al. [17]	^50%^
Hemamy M et al. [18]	^66.66%^
Khaksarian M et al. [19]	^83.33%^
Khoshbakht Y et al. [20]	^66.66%^
Wang LJ et al. [21]	^50%^
Kean JD et al. [22]	^100%^
Pelsser L et al. [23]	^50%^
Dolp A et al. [24]	^83.33%^
Kirkland AE et al. [25]	^83.33%^

Result

**Figure 1:**
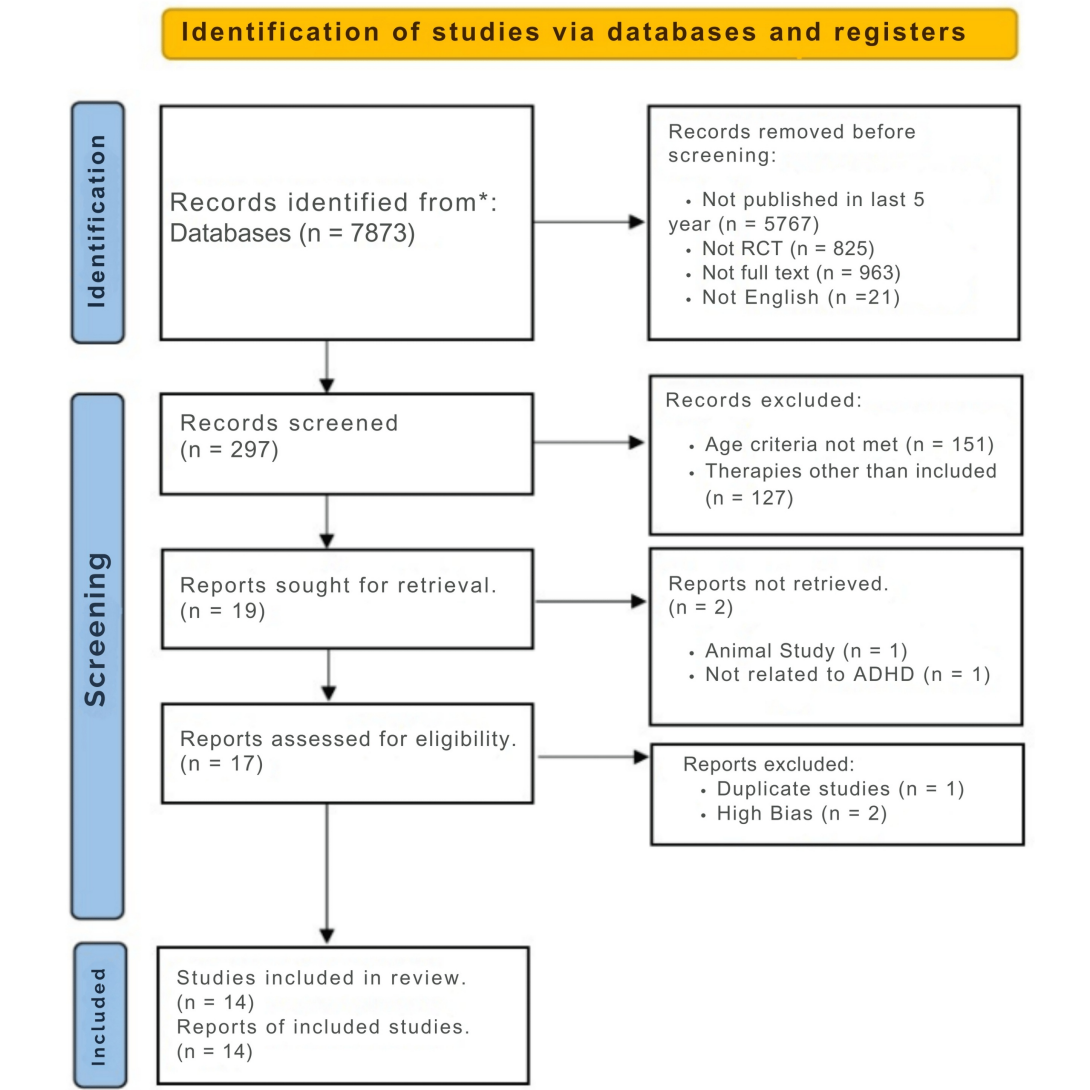
PRISMA flowchart PRISMA: Preferred Reporting Items for Systematic Reviews and Meta-Analyses

As outlined in the methodology, we utilized multiple search engines and focused on reputable databases, such as PubMed, primarily using the specified keywords. Our initial search yielded a total of 7873 studies. By applying filters based on publication timeframe, language, study design, and full-text availability, we narrowed down the results to 297 records. Duplicates and studies lacking the specified dietary interventions were subsequently excluded. After thorough review and discussions among co-authors, 16 studies remained for quality appraisal, with 14 ultimately meeting the inclusion criteria after full-text assessment. Please refer to Figure 1 for the PRISMA chart detailing the selection process.

The data collected from the included studies are given in Table 4.

**Table 4:** Results AFC refers to artificial food colors used in food and beverages. MPH stands for Methylphenidate, a medication commonly used to treat attention deficit hyperactivity disorder (ADHD). DASH diet represents dietary approaches to stop hypertension, a dietary plan designed to prevent and control high blood pressure. Bf denotes Bifidobacterium bifidum, a type of beneficial bacteria found in the gut.

Study number as per references given below	Year of publication	Total no. of participants	Mean age	Cohort 1/Group 1/Control (intervention was given in this case)	Cohort 2/Test Group (intervention was given in this group)	Effect on symptoms in Cohort 1	Effect on symptoms in Cohort 2	No. of dropouts
Stevens AJ et al. [10]	2019	18	7-12, 10	Participants in a normal double-blind study were randomly allocated to receive a placebo.	Children were provided dietary micronutrients to conduct a fecal microbiota sequencing analysis.	Children in the placebo group did not improve.	Participants who received micronutrients had lower levels of Bifidobacterium species in their feces, along with improved focus and general function.	1
Hemamy M et al. [12]	2021	66	6-12, 9	Children received a placebo.	Magnesium and vitamin D supplements were given to children.	Children who were given a placebo did not improve.	Magnesium and 25-hydroxyvitamin D3 blood levels were higher in the intervention group. Additionally, emotional states and social indices improved.	0
Rucklidge JJ et al. [14]	2021	89	7-12, 9.5	Children received a placebo.	Children were provided with micronutrients.	The placebo group showed a lesser impact on symptoms.	Micronutrients increased overall performance, reduced impairment, and enhanced aggression and emotional regulation, but not impulsivity or hyperactivity.	4
Samadi M et al. [15]	2022	86	6-12, 9	Children received a placebo.	Vitamin D3 (2000 IU/day) was administered to children.	TNF- and IL-6 levels did not decrease.	The vitamin D cohort experienced substantially higher serum concentrations of 25(OH) D. Serum TNF- and IL-6 levels did not decrease.	11
Honarvar NM et al. [16]	2022	86	6-12, 8	Children received a placebo.	Vitamin D3 was administered to children (2000 IU).	Increase in the circulating active form of vitamin D and no decrease in the 8-isoprostan level.	There are no significant findings in kids with vitamin D supplementation, as indicated by no reduction in TAC (total antioxidant capacity), 25-OHD, or 8-isoprostan levels.	11
Canal LA et al. [17]	2019	6	9-13, 11	No control arm.	A four-month gluten-free diet.	—	50% showed improvement in ADHD symptoms.	—
Hemamy M et al. [18]	2020	74	Intervention group: 9.06 ± 1.76, control group: 9.15 ± 1.46	Placebos with Mg2+ and calciferol.	Children received calciferol and Mg2+.	There were no notable changes.	Providing both vitamin D and magnesium improved their responses to anxiety-inducing issues, but it had very little effect on psychosomatic problem ratings.	8
Khaksarian M et al. [19]	2021	90	6-16, 11	20 or 30 mg/d of MPH.	20 or 30 mg/d of MPH and saffron capsule.	Reduced ADHD symptoms.	Compared to individual treatments, the use of MPH and saffron in the management of this disorder was more successful.	20
Khoshbakht Y et al. [20]	2021	80	6-12, 9	Children received a controlled diet.	DASH diet.	Children on the control diet showed no improvement compared to children who received the DASH diet.	The symptoms of ADHD may be reduced by following a DASH-style diet. The current findings require further RCTs with participants of both sexes and longer follow-up times.	0
Wang LJ et al. [21]	2022	30	6.9	A single-arm, open-label clinical trial with no control arm.	Bf-688 sachet.	—	With regard to ADHD, clinical symptoms improved. The supplement Bf-688 also considerably changed the composition of the gut flora.	0
Kean JD et al. [22]	2017	144	8.7	Children received a placebo for 14 weeks.	Marine oil extract (PCSO-524®) was given to children for 14 weeks.	The placebo group did not improve.	The findings indicate that PCSO-524® may help youngsters with both clinical and subclinical signs of ADHD by lowering their levels of hyperactivity and inattention.	32
Pelsser L et al. [23]	2020	61	2–16, (9)	—	The study included all kids who started the few-foods diet during a three-month period in three facilities with specialized medical care. Clinical responders who experienced behavioral improvements of at least 40% started the reintroduction phase.	—	Among the participants, 48% of children were taking medication at the beginning of the study, and 70% of those who were not on medication initially showed behavioral improvements of 40% or more. A total 72% reduction in symptoms.	4
Dolp A et al. [24]	2020	10	8-14, 11	There was no control group or blinding in this study.	Participants underwent a four-week oligoantigenic diet.	—	62.5% of the children were judged as responding to the diet by two blinded raters and one non-blinded rater; however, one blinded rater disagreed with the findings of one case and rated 50% of the children as responding.	—
Kirkland AE et al. [25]	2020	18	Young adults are mentioned in the article without age.	Children without ADHD were given either 225 mg of artificial food coloring disguised as chocolate cookies or a placebo.	Children with ADHD were assigned either to receive 225 mg of artificial food coloring cloaked in chocolate biscuits or a placebo.	The extended control group didn't experience any consequences from AFC.	Mild signs of inattention were present.	—

Discussion

Gluten-Free Diet

Canal LA et al. conducted a pilot study involving six ADHD-diagnosed patients aged 9 to 13. Over four months, participants adhered to a gluten-free diet while undergoing clinical assessments. Despite negative celiac serology, all reported significant alleviation of digestive symptoms and headaches. While three patients noted ADHD symptom improvements, CPT-II scores showed no significant change, underscoring the potential benefits of gluten-free diets in managing ADHD symptoms and addressing non-celiac gluten sensitivity [17].

Vitamin and Mineral Supplements

Hemamy M et al. conducted a double-blind trial with 66 children administering vitamin D and magnesium supplements over 56 days. Compared to placebo, significant increases in serum Mg2+ and calciferol levels were observed in the intervention group. Although improvements were noted in social and anxiety-related symptoms, psychological aspects showed no significant changes [18]. Similarly, Honarvar NM et al. investigated vitamin D3 supplementation in ADHD children aged 6-12 over three months, revealing no significant effects on antioxidant markers compared to placebo [16]. Khaksarian M et al. explored the combined effects of methylphenidate (MPH) and Crocus sativus (saffron) in treating ADHD in 70 children aged 6-16 years. Based on BMI, participants were randomly assigned to receive either MPH alone or MPH combined with saffron. Both groups showed reduced ADHD symptoms after eight weeks, with the MPH-saffron group demonstrating significantly lower ADHD Rating Scale-IV scores compared to the MPH group at four weeks, suggesting potential synergistic effects of saffron and MPH in managing ADHD symptoms [19]. Hemamy M et al. further investigated Mg2+ and vitamin D supplementation in 66 ADHD adolescents, noting significant increases in serum magnesium and 25-hydroxyvitamin D3 levels after eight weeks of intervention [12]. These increases correlated with improved behavioral and emotional health, suggesting the potential benefits of mineral supplementation in managing ADHD symptoms.

Dietary Approaches

Khoshbakht Yadollah et al. examined the dietary approaches to stop hypertension (DASH) diet in 86 children aged 6-12 years and diagnosed with ADHD over three months. Significant improvements in ADHD symptoms were reported compared to a control diet, highlighting the potential role of dietary interventions in symptom management and nutrient intake [20]. Pelsser L et al. investigated a few foods diet (FFD) in children with ADHD, reporting that 78% of participants discontinued medication while on the diet. The study emphasized the potential benefits of dietary interventions in managing ADHD symptoms, suggesting that dietary factors play a crucial role in symptomatology [23]. Dölp A et al. explored an oligoantigenic diet (OD) in 10 children with ADHD, finding significant symptom improvements after the intervention, indicating the relevance of dietary intolerances in managing ADHD symptoms [24].

Other Dietary Interventions

Kirkland AE et al. examined the impact of artificial food colorants (AFC) on ADHD symptoms and EEG in college students [25]. Despite symptom reductions post-AFC exposure, EEG and focus tests showed no significant differences compared to placebo, indicating the potential behavioral impacts of dietary components on ADHD symptoms.

Probiotics and Gut Microbiota

Wang LJ et al. investigated Bifidobacterium bifidum supplementation in reducing ADHD symptoms and altering gut microbiota composition, demonstrating positive effects on symptoms and suggesting a potential role for probiotics in managing ADHD [21]. Stevens AJ et al. found that changes in ADHD symptoms were associated with alterations in gut microbiota following vitamin supplementation. Specifically, participants receiving micronutrients showed decreased levels of Bifidobacterium species in their feces, alongside improvements in focus and overall functioning [10].

Marine Oils

Kean JD et al. studied PCSO-524® from marine oils in 144 individuals with severe ADHD symptoms, reporting significant improvements in inhibition and reduced error-making compared to placebo, suggesting the potential benefits of marine oils in managing ADHD symptoms [22].

This systematic review synthesizes evidence from various studies on dietary interventions and supplements for managing ADHD symptoms. Key findings include the potential efficacy of gluten-free diets, the DASH diet, and specific supplements like vitamin D and magnesium in alleviating symptoms. Additionally, unconventional treatments, such as saffron combined with methylphenidate and marine oil extract (PCSO-524®), show promise in improving ADHD-related behaviors.

The findings underscore the significant impact of dietary factors on ADHD symptomatology, suggesting that personalized dietary interventions could complement traditional therapies. Gluten-free and nutrient-rich diets appear beneficial, possibly by reducing inflammation and addressing nutritional deficiencies in ADHD. Moreover, supplements and novel treatments like probiotics offer potential avenues for enhancing symptom management through gut-brain axis modulation.

These results highlight the importance of considering dietary modifications as part of ADHD treatment strategies. By understanding how specific dietary components influence neurodevelopmental processes, clinicians can offer more comprehensive care tailored to individual needs. Integrating dietary assessments and interventions into clinical practice may improve treatment outcomes and reduce reliance on pharmacotherapy alone.

Limitations

The variability in study methodologies, participant demographics, and outcome measures limits the generalizability of findings. Additionally, the short-term nature of many studies precludes definitive conclusions about the long-term effectiveness and sustainability of dietary interventions. Furthermore, interactions between dietary modifications and traditional ADHD medications require further investigation to optimize combined therapeutic approaches.

## Conclusions

This systematic review addresses the efficacy of various dietary interventions and supplements in managing ADHD symptoms, aiming to synthesize current evidence and identify promising approaches to complement traditional therapies. The review highlights significant findings across different dietary interventions and supplements. Key interventions include gluten-free diets, which show potential in alleviating ADHD symptoms despite negative celiac serology, and vitamin and mineral supplements like vitamin D and magnesium, demonstrating varying degrees of efficacy in improving symptomatology. Novel treatments, such as saffron combined with methylphenidate and marine oil extracts, also show promise in enhancing ADHD-related behaviors. Additionally, dietary approaches, such as the DASH diet, and specific exclusions like the few foods diet and oligoantigenic diet underscore the role of nutrition in symptom management. In conclusion, integrating dietary assessments and interventions into ADHD treatment strategies holds promise for improving outcomes and providing more comprehensive care tailored to individual patient needs.
